# Arbovirus Prevalence in Mosquitoes, Kenya

**DOI:** 10.3201/eid1702.091666

**Published:** 2011-02

**Authors:** A. Desiree LaBeaud, Laura J. Sutherland, Samuel Muiruri, Eric M. Muchiri, Laurie R. Gray, Peter A. Zimmerman, Amy G. Hise, Charles H. King

**Affiliations:** Author affiliations: Children’s Hospital Oakland Research Institute, Oakland, California, USA (A.D. LaBeaud);; Case Western Reserve University, Cleveland, Ohio, USA (A.D. LaBeaud, L.J. Sutherland, L.R. Gray, P.A. Zimmerman, A.G. Hise, C.H. King);; Ministry of Public Health and Sanitation, Nairobi, Kenya (S. Muiruri, E.M. Muchiri)

**Keywords:** Arboviruses, zoonoses, viruses, Rift Valley fever virus, West Nile virus, vectors, mosquitoes, RT-PCR, Kenya, research

## Abstract

Few studies have investigated the many mosquito species that harbor arboviruses in Kenya. During the 2006–2007 Rift Valley fever outbreak in North Eastern Province, Kenya, exophilic mosquitoes were collected from homesteads within 2 affected areas: Gumarey (rural) and Sogan-Godud (urban). Mosquitoes (n = 920) were pooled by trap location and tested for Rift Valley fever virus and West Nile virus. The most common mosquitoes trapped belonged to the genus *Culex* (75%). Of 105 mosquito pools tested, 22% were positive for Rift Valley fever virus, 18% were positive for West Nile virus, and 3% were positive for both. Estimated mosquito minimum infection rates did not differ between locations. Our data demonstrate the local abundance of mosquitoes that could propagate arboviral infections in Kenya and the high prevalence of vector arbovirus positivity during a Rift Valley fever outbreak.

Emerging zoonotic diseases threaten the health and security of human and animal populations throughout the world (*1*). Because arthropod-borne viruses, or arboviruses, can be spread by competent mosquito vectors across great distances, they pose substantial risk to other regions in which the disease is currently nonendemic (*1*). Zoonotic arboviruses circulate in sylvatic and peridomestic cycles involving wild animals and nearby humans. Often these arboviruses remain undetected by health care systems (*2**–**4*). Kenya has had multiple arbovirus outbreaks in the past 2 decades resulting in economic and public health distress, including yellow fever in 1992 (*5**,**6*) and 1995 (*7*), chikungunya fever in 2004 (*8*), and Rift Valley fever (RVF) in 1997 (*9*) and 2006 (*10*). Much remains unknown about the true prevalence of arboviruses in Kenya and the mosquito vectors responsible for virus maintenance and transmission. We investigated the local abundance of mosquitoes in Kenya that are infected with RVF virus (RVFV) and West Nile virus (WNV); mosquitoes were collected near human habitation during a period of prolonged heavy rainfall.

*Rift Valley fever virus*, family *Bunyaviridae*, genus *Phlebovirus*, is a vector-borne virus endemic to Africa and the Middle East (*11*). Recent outbreaks of RVF have resulted in substantial human illness and livestock losses in Kenya (*9**,**10**,**12*). Domestic ungulates are a principal source of transmissible RVFV, and human infection has been associated with direct animal contact, specifically with cattle, sheep, and goats (*2**,**9**,**12*). It is unclear which, if any, animal species maintain RVFV during interepidemic periods, and it is possible that RVFV is maintained solely within arthropod vectors during these periods (*13*).

*West Nile virus*, family *Flaviviridae*, genus *Flavivirus*, is a vector-borne virus that is maintained in nature between mosquitoes and birds (*11*). Humans and other mammals are incidental hosts and do not play a role in the natural preservation of WNV (*11*). Because most WNV infections are self-limiting and subclinical, human infections in Kenya are often misdiagnosed (*14*). As a result, the true prevalence of WNV in the country is probably underestimated (*15*). Further clarification of the true presence and circulation of WNV in mosquito vectors could enhance human WNV case detection in the region.

Few studies have investigated the many mosquito species that harbor arboviruses in Kenya (*16**–**21*). Entomologic surveys have demonstrated that mosquitoes that usually facilitate outbreaks of arboviral diseases, specifically *Aedes* spp., *Anopheles* spp., and *Culex* spp., flourish in Kenya (*16**,**18**,**19**,**22**–**26*). At least 40 different mosquito species can harbor RVFV, although their ability to transmit RVFV varies (*14**,**20**,**21**,**27**–**29*). Furthermore, although many species are susceptible to RVFV infection, studies of mosquito vectors in northeastern Kenya have shown that the proportion of positivity in individual species differs greatly (5.9% *An. squamosus*, 30% *Ae. ochraceus*, 42% *Ae. mcintoshi*) (R. Sang, pers. comm.). RVFV can also be transovarially transmitted in at least 1 mosquito species, *Ae. mcintoshi* (*17*). The isolation of WNV from a non–blood-feeding male *Cx. univattatus* mosquito trapped in northwestern Kenya indicates that WNV also transmits transovarially in that region (*23*).

## Materials and Methods

### Sampling

To evaluate the temporal profile of vector mosquitoes in North Eastern Province, Kenya, trapping was performed during the dry season (August 2006) and during the rainy season (December 2006–January 2007). Mosquitoes collected during December 2006 and January 2007 were trapped during an epizootic/epidemic of RVF. Homestead trapping locations adjacent to homesteads in the regions were randomly selected from previously prepared census lists and were restricted to only those homes where animals (cows, goats, or sheep) were housed alongside human habitats. Each household had only 1 CDC light trap (John W. Hock Company, Gainesville, FL, USA) located next to animal structures; trap was set 1 time for 12 hours, 6:00 pm–6:00 am.

**Figure 1:**
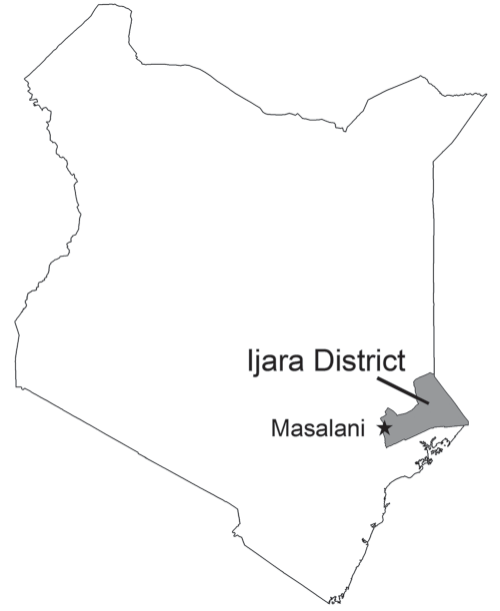
Location of Masalani Division of Ijara District, North Eastern Province, Kenya.

Mosquito sampling was conducted in 2 areas within Masalani Division, Ijara District, North Eastern Province, where human surveillance had taken place 8 months before the RVF outbreak (*2*) (Figure 1). Traps were located in the rural village of Gumarey (1°40′12′′S, 40°10′48′′E) and the town of Sogan-Godud (1°41′24′′S, 40°10′12′′E). The population of Gumarey consists of seminomadic herders who live in traditional grass huts near their livestock. Sogan-Godud is more urban with a marketplace and contains a greater proportion of tin-roofed permanent dwellings. The centroids of these 2 locations are 5 km apart, and the borders are within 500 m of each other. Both locations had persistent local flooding during the extensive El Niño/Southern Oscillation associated heavy rains during 2006–2007, and both are within 10 km of the Tana River. Persons seropositive for RVFV from both locations were documented in early 2006; seroprevalence rates were greater in rural Gumarey (20% vs. 6%) (*2*). During that initial study, all homesteads were identified and their locations identified by Global Positioning Satellite. Spatially referenced data on individual residence and homestead exposure features were maintained and analyzed by using ArcGIS version 9.2 (ESRI, Redlands, CA, USA).

### Mosquito Preparation

Mosquito genera were identified in Kenya by local entomologists on the basis of microscopic morphologic appearance. Only female mosquitoes were included in this study; male mosquitoes were not further tested. Single leg specimens were preserved in RNAlater (Ambion, Austin, TX, USA) and transported to Case Western Reserve University (Cleveland, OH, USA) for processing. DNA and RNA were extracted from mosquito legs by using a column purification kit (QIAGEN, Valencia, CA, USA) with the following modifications: each mosquito leg was placed into a microcentrifuge tube containing 150 μL of RNeasy lysis buffer and finely ground with a disposable RNase/DNase-free pestle. After homogenization, samples were processed according to established protocols through either individual QIAGEN RNeasy columns or 96-well plates, washed, and eluted in RNase-free water. The DNase step was omitted so that DNA and RNA could be collected from samples. Individual RNA samples were combined in pools of <12 mosquitoes (median 10, mean 8.7), based on homestead trap for cDNA synthesis and PCR or quantitative reverse transcription–PCR (qRT-PCR).

### Primers and Generation of Standard Controls

To verify the quality of the RNA and the integrity of the cDNA products after reverse transcription, mosquito 18S rRNA primers were designed to amplify within a region conserved in many *Culicidae* spp. mosquitoes (*30*). These mosquito primers were designed against the 18S rRNA gene sequences for *Aedes* spp. (GenBank accession no. AB085210) and *Culex* spp. (GenBank accession no. U48385) mosquitoes to amplify an optimally sized product (124 bp) for qRT-PCR. WNV primers were based on the New York 1999 WNV isolate (GenBank accession no. AF196835.2) described by Lanciotti et al. (*11*). These primers have been shown to detect Old and New World WNV strains, including a strain isolated in Kenya in 1998 (*11**,**31*). RVFV primers, which amplify a conserved region of the large segment (90 bp), were used as described by Bird et al. (*32*) (Table 1).

**Table 1:** Oligonucleotide primer pairs used in assay during Rift Valley fever outbreak, Kenya, 2006–2007*

Target	Forward sequence, 5′ → 3′	Reverse sequence, 5′ ← 3′	Product size, bp	GenBank accession no.
Mosquito 18S rRNA	GATCAAGTGGAGGGCAAGTC	AAGGAGTAGCACCCGTGTTG	124	AB085210.1
RVFV	TGAAAATTCCTGAGACACATGG	ACTTCCTTGCATCATCTGATG	90	DQ375404.1
WNV	CAGACCACGCTACGGCG	CTAGGGCCGCGTGGG	103	AF196835.2

*RVFV, Rift Valley fever virus; WNV, West Nile virus.

An RVFV standard control was generated by amplifying RVFV vaccine strain rMP-12 in Vero E6 cells for 72 h and then extracting viral RNA from supernatant and cell lysate by using the PureLink Total RNA Purification System (Invitrogen, Carlsbad, CA, USA). A WNV standard control was generated by using confirmed WNV-positive samples received from the Ohio Department of Health. Mosquito 18S rRNA-, WNV-, and RVFV-positive controls were generated by using the primers listed in Table 1 and cloned by using the pCR 8/GW/TOPO TA cloning kit (Invitrogen). All inserts were verified by sequencing of the plasmids.

### cDNA Synthesis, PCR, and qRT-PCR Conditions

Two-step qRT-PCR was performed on all pooled samples. First-step total cDNA synthesis was performed on RNA extracted from mosquito leg tissue by using random hexamer primers. The reaction mixture was incubated at 65°C for 5 min, chilled on ice, and combined with 4 μL 5× First-Strand Buffer, 1 μL 0.1M dithiothreitol, 1 μL RNase inhibitor, and 0.5 μL SuperScript III Reverse Transcriptase (Invitrogen). The final reaction mixture was incubated at 25°C for 10 min, 50°C for 50 min, and heat inactivated at 70°C for 15 min.

**Figure 2:**
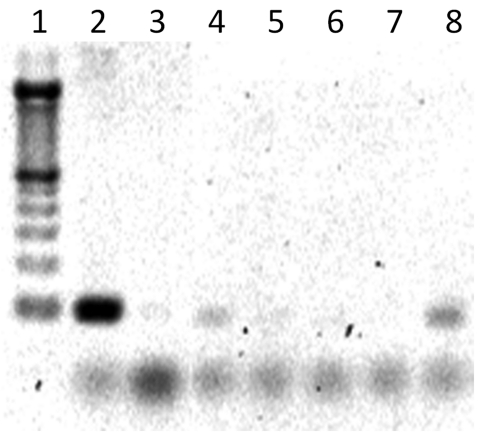
PCR gel showing positive Rift Valley fever virus bands (90 bp). Lane 1, molecular mass ladder; lane 2, Rift Valley fever virus MP-12 positive control; lane 3, negative control; lane 4, pool 103 (positive); lane 5, pool 86 (negative); lane 6, pool 104 (negative); lane 7, pool 87 (negative); lane 8, pool 105 (positive).

After cDNA synthesis, 1 μL of total cDNA was added to the qRT-PCR mixture containing 0.2 μmol/L forward primer and 0.2 μmol/L reverse primer (18S and WNV testing), 12 μL FastStart Universal SYBR Green Master mix (Roche, Indianapolis, IN, USA), and 12 μL sterile, nuclease-free water. The qRT-PCR was conducted in an Applied Biosystems 7300 instrument (Applied Biosystems, Foster City, CA, USA) with a heating cycle of 50°C for 2 min and 95°C for 10 min; followed by 45 cycles of 95°C for 15 s, 60°C for 1 min, 95°C for 15 s; and an additional dissociation step of 60°C for 1 min. All samples, which registered a cycle threshold value <35 cycles and had a lower cycle threshold value than negative controls, were considered positive for their respective targets. All pools were further PCR tested for RVFV by using 2 μL cDNA, 0.5 μmol/L each forward/reverse RVFV primer, 10.5 μL sterile, nuclease-free water, and 12.5 μL JumpStart ReadyMix Taq (Sigma-Aldrich, St. Louis, MO, USA). PCR cycling parameters were 94°C for 5 min, with 30 cycles of 95°C for 30 s, 60°C for 1 min, 72°C for 1 min, and a 10-min 72°C extension. PCR products were run on a 2% agarose gel with SYBR Safe (Invitrogen) for band visualization (Figure 2). An initial sampling of RVFV PCR products was cloned by using the above-mentioned methods and sequenced for confirmation. Mosquito minimum infection rates (MIR) for RVFV and WNV were calculated on the basis of maximum-likelihood estimation by using the PoolScreen 2.0 program (University of Alabama at Birmingham, Birmingham, AL, USA) (*33**–**35*).

## Results

A total of 74 trapping events occurred at 38 different homestead locations in the study villages. Because of the annual drought, no mosquitoes were recovered in the traps set in August. Overall, 12,080 mosquitoes were collected: 9,701 mosquitoes during the 7 trapping nights in December (December 12–19, 2006) and 2,379 mosquitoes during the 6 trapping nights in January (January 19–26, 2007). The most abundant mosquitoes trapped were of the genus *Culex*. For the entire trapping period 7,853 *Culex* spp., 3,488 *Anopheles* spp., 682 *Mansonia* spp., and 57 *Aedes* spp. mosquitoes were trapped and identified. Traps caught an average of 199 mosquitoes per trap, with an average of 141 *Culex* spp. mosquitoes.

**Figure 3:**
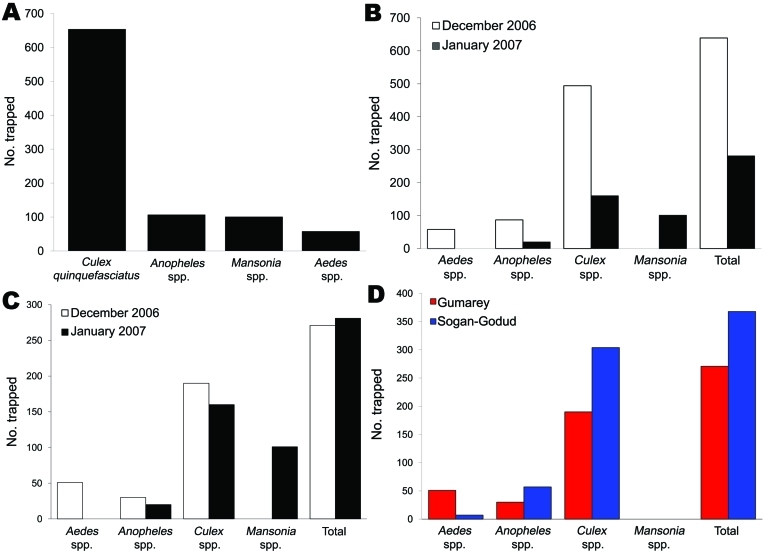
Identification of mosquitoes trapped, Gumarey and Sogan-Godud, Masalani Division of Ijara District, Kenya, 2006–2007. A) Mosquito species trapped during sampling effort. B) Mosquitoes trapped by date. *Aedes* spp. mosquitoes were found in traps only in December 2006 and *Mansonia* spp. mosquitoes only in January 2007. C) Temporal comparison of mosquitoes trapped in Gumarey. D) Mosquitoes trapped by study area, December 2006.

To estimate location-specific risk for arbovirus transmission during the December 2006–January 2007 sampling period, 920 mosquitoes collected in the field were pooled for PCR detection of RVFV and WNV. These 920 exophilic mosquitoes were trapped at 30 different homesteads adjacent to animal structures, yielding 105 pools based on trap location (homestead) per trapping night with an average of 10 mosquitoes (range 1–12 mosquitoes) per pool. In 23 Gumarey homesteads, 552 mosquitoes were trapped and divided into 65 total pools in the laboratory (1–12 individual mosquito legs/pool, based on trap night). A total of 368 mosquitoes were trapped at 7 Sogan-Godud homesteads and divided into 40 pools for testing (1–11 individual mosquito legs/pool, based on trap night). Most mosquitoes tested were morphologically identified as C*ulex* spp. (n = 654, 71%) (Figure 3). The remaining mosquitoes were identified as *Anopheles* spp. (n = 107, 12%), *Mansonia* spp. (n = 101, 11%), and *Aedes* spp. (n = 58, 6%). Synthesis of total cDNA was successful; 99% of samples amplifyed 18S, and the remaining 1% was removed from further testing.

In total, of the 105 trap-night pools, 18% (95% confidence interval [CI] 11.3%–26.8%) had positive results by PCR for WNV and 22% (95% CI 14.5%–31.1%) for RVFV (Table 2). Of the 65 pools from Gumarey, 14% (95% CI 6.5%–24.7%) and 30% (95% CI 18.6%–41.8%) had positive results for WNV and RVFV, respectively. Of the 40 pools from Sogan-Godud, 25% (95% CI 12.7%–41.2%) and 10% (95% CI 2.8%–23.7%) had positive results for WNV and RVFV, respectively. A comparison of positive results for RVFV in mosquito pools across villages was significantly different (p = 0.0279); a comparison of positive results for WNV across village pools was not (p = 0.1932). Three percent of mosquito pools tested had positive results for both WNV and RVFV.

**Table 2:** PCR results for RVFV and WNV in mosquito pools, by pool, Kenya, 2006–2007*

Virus and location	No. positive/ no. tested (%)	MIR estimate, %	95% CI
Total			
WNV	19/105 (18)	2.3	1.3–3.6
RVFV	23/105 (22)	2.8	1.7–4.2
Gumarey			
WNV	9/65 (14)	1.8	0.75–3.40
RVFV	19/65 (30)	3.9%	2.3–6.3
Sogan-Godud			
WNV	10/40 (25)	3.0%	1.4–5.7
RVFV	4/40 (10)	1.1%	0.29–2.90

*RVFV, Rift Valley fever virus; WNV, West Nile virus; MIR, minimum infection rate; CI, confidence interval.

**Figure 4:**
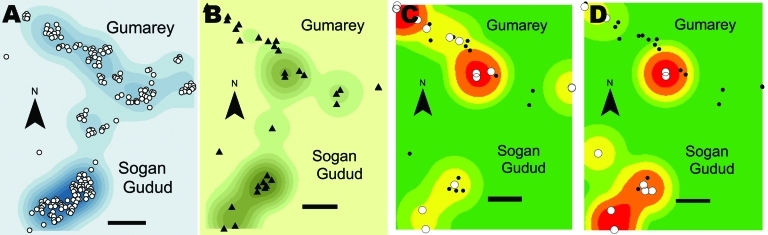
Distribution of human population and infected and uninfected mosquitoes across the selected study areas, Gumarey and Sogan-Godud, Masalani Division of Ijara District, Kenya. A) Area homestead locations (circles) and relative area density of human population (contours, 500-m kernel density; darker color indicates higher values). B) Study trap locations (triangles) and area density of mosquitoes (contours for average mosquitoes per trap, 500-m kernel density). C) Homestead locations of mosquito pools testing positive (white circles) and negative (black circles) for Rift Valley fever virus. Relative local density of positive pools per 500 m is indicated by contours. D) Homestead locations of mosquito pools testing positive (white circles) and negative (black circles) for West Nile virus. Relative density of positive pools is indicated by contours.

Figure 4 shows the area distribution of homesteads, mosquito traps, and local abundance of RVFV-positive and WNV-positive trap pools. When analyzed based on the 30 homestead locations, 10 (33%; 95% CI 17.3%–52.8%) homesteads with tested mosquitoes were positive for WNV, versus 15 (50%; 95% CI 31.3–68.7%) for RVFV (Table 3). Most (5/7; 71%) Sogan-Godud homesteads were positive for WNV (95% CI 29.0%–96.3%), compared with 5/23 (22%; 95% CI 7.5%–43.7%) for Gumarey, although MIRs did not differ (Table 3). Homestead WNV positivity significantly differed between villages (p = 0.0256); RVFV positivity of homesteads did not (p = 1.000). RVFV homestead positivity rates were similar between the 2 locations; 12/23 (52%; 95% CI 30.6–73.2) mosquito pools in Gumarey homesteads had positive results, versus 3/7 (43%; 95% CI 9.9–81.6) in Sogan-Godud.

**Table 3:** PCR results for RVFV and WNV in mosquito pools, by homestead source, Kenya, 2006–2007*

Virus and location	No. positive/ no. tested (%)	MIR estimate, %	95% CI
Total			
WNV	10/30 (33)	2.0	0.87–4.20
RVFV	15/30 (50)	4.6	2.2–8.9
Gumarey			
WNV	5/23 (22)	1.4	0.39–3.30
RVFV	12/23 (52)	5.3	2.4–1.0
Sogan-Godud			
WNV	5/7 (71)	7.5	1.4–24
RVFV	3/7 (43)	2.4%	0.37–12.00

*RVFV, Rift Valley fever virus; WNV, West Nile virus; MIR, minimum infection rate; CI, confidence interval; HH, homestead.

**Figure 5:**
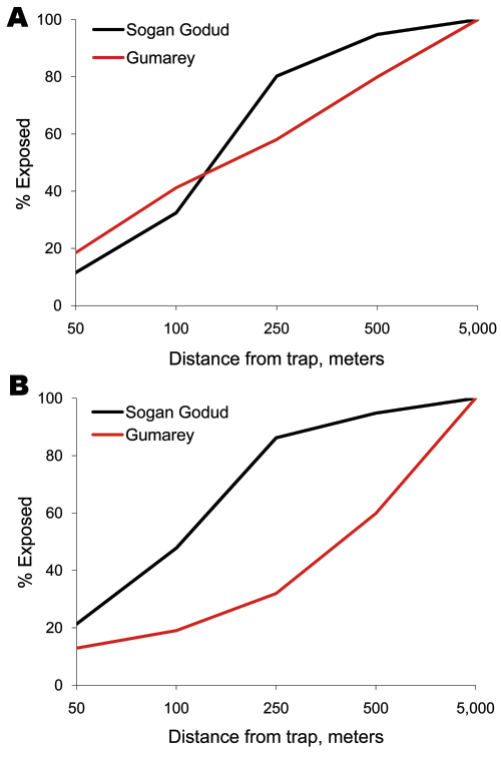
Cumulative proportion of residents within range of Rift Valley fever virus (RVFV)–positive (A) and West Nile virus (WNV)–positive (B) mosquito pools, by village, Gumarey and Sogan-Godud, Masalani Division of Ijara District, Kenya.

In terms of the general population, by using geographic information systems analysis of spatially referenced census data (Figure 5), we confirmed that >30% of Sogan residents and >40% of Gumarey residents lived within 100 meters of an identified RVFV-positive mosquito trap site. Forty-eight percent of Sogan residents lived within 100 meters of a WNV-positive trap site; only 19% of Gumarey residents lived within 100 meters of a WNV-positive site.

Of the mosquitoes trapped during this study, *Culex* spp. was the predominant genus, although *Aedes* spp., *Anopheles* spp., and *Mansonia* spp. mosquitoes were also recovered during nocturnal light trapping. In the pools that contained only 1 genus of mosquito, positivity varied. A total of 63 pools were composed solely of *Culex* spp. mosquitoes (specifically *Cx. quinquefasciatus*), 9 of which were positive for RVFV. Additionally, 1 of 4 pools containing only *Aedes* spp. mosquitoes were RVFV positive, 3 of 8 *Anopheles* spp.–only mosquito pools had positive results for RVFV, and 3 of 8 *Mansonia* spp.–only mosquito pools had positive results for RVFV. WNV-positive pools composed of only 1 genus included 3 of 4 *Aedes* spp.–only mosquito pools and 15 of 63 *Culex* spp.–only mosquito pools. All *Aedes* spp. mosquitoes collected were trapped in December but were absent in the traps in January (Figure 3). The temporal distribution of these mosquitoes correlates with previous studies showing that *Aedes* spp. mosquitoes predominate in the initial weeks after substantial flooding and then curtail after the first month of flooding, at which time *Culex* spp. and *Anopheles* spp. mosquitoes emerge as the predominant species (*17**,**36*). It is believed that the dramatic proliferation of transovarially infected *Aedes* spp. mosquitoes immediately after flooding re-introduces virus into an epizootic/epidemic cycle, after which *Culex* spp. mosquitoes propagate the virus in an epizootic/endemic cycle among humans and animal species (*17*).

## Discussion

A substantial proportion of the mosquito population collected within our study area consisted of RVFV- or WNV-infected potential vectors. The close proximity of these infected mosquitoes to amplifying hosts and susceptible animals and humans during an RVFV epizootic/epidemic warrants further investigation of transmission dynamics. RVFV RNA in mosquitoes collected within the area was high, and the substantial presence of WNV RNA in these mosquito samples was unexpected. The presence of WNV in mosquitoes from Sogan-Godud and Gumarey in our study corroborates recent documentation of the widespread presence of WNV in Kenya and the ability of mosquito populations, including *Cx. quinquefasciatus*, to acquire and transmit WNV (*23*).

The previous isolation of WNV from male *Culex* spp. mosquitoes in Rift Valley Province suggests a natural transovarial transmission cycle among some mosquito vectors but is unlikely to contribute greatly to virus maintenance between enzootic periods (*23*). Additionally, although human epidemics and outbreaks of WNV have not been reported, the presence of the virus in local mosquitoes suggests that the virus is maintained in a natural cycle yet to be elucidated and that the actual incidence of WNV in human populations in the region could be underestimated. Improved field diagnostics are necessary for rapid and accurate diagnosis of circulating arbovirus threats and expedient translation into preventive public health practices.

The isolation of RVFV and WNV RNA from mosquito leg samples confirms that these viruses were disseminated within the bodies of the mosquitoes tested. These results also confirm that single mosquito leg samples are sufficient for PCR/qRT-PCR detection of RVFV and WNV, respectively. Positive results from testing of the mosquito legs also diminish concern about false-positive results from testing whole mosquitoes, which might contain recent bloodmeals with substantial viral content. Our study confirms that RVFV disseminates to the legs of wild *Cx. quinquefasciatus* mosquitoes and suggests that these mosquitoes, promiscuous feeders, could play a role in the maintenance or transmission of RVFV in disease-endemic regions (*20**,**21*). Other vector competence studies have shown that RVFV does disseminate in *Cx. quinquefasciatus* mosquitoes but have yet to show that they are efficient vectors for RVFV (*20**,**21**,**37*). Although identification of viral RNA in the legs of *Cx. quinquefasciatus* as well as the other mosquitoes tested supports dissemination of virus, no conclusions can be made from these results regarding the role of these mosquitoes in maintaining these arboviruses in this environment or their ability to transmit virus. Additional studies are required to determine vector competence of *Cx. quinquefasciatus* and other mosquito species tested for these 2 viruses.

During this RVFV outbreak, we documented >1 arbovirus circulating in local mosquitoes. During an arbovirus outbreak, other viruses may be circulating concomitantly without recognition and serve as alternative causes of fever. Additional arthropod surveillance studies during RVFV outbreaks in Kenya have found arboviruses in mosquitoes, including flaviviruses and alphaviruses, which can cause febrile illness in humans (*38*). Because diseases from arboviral infections can be nonspecific in humans and animals, it is necessary, even during large outbreaks, to document the true cause of disease with detailed testing. Cases of other arboviral infections could be missed if suspected cases are attributed to the epidemic arbovirus without accurate diagnosis.

Although MIRs for RVFV were similar in the 2 villages studied, rural Gumarey was more likely to have RVFV-positive pools than was Sogan-Godud. This finding concurs with previous human seroprevalence studies that found that risk for being RVFV seropositive is 4× greater for those living in Gumarey than for those in Sogan-Godud (*2*). Gumarey residents were more likely to report greater contact with animals and mosquitoes (*2*). Continued research to identify village level and landscape factors responsible for increased human transmission is necessary. Although RVFV can be transmitted to humans by the bite of an infected mosquito, alternative forms of human exposure, such as aerosol and direct contact, may be more critical for transmission during epidemics (*2**,**28**,**36*). More research must be conducted to elucidate the most common and most effective routes of RVFV transmission to humans during epidemic and interepidemic periods.

Few research studies have documented the presence of WNV and the vectors responsible for its transmission in Kenya. The identification of WNV in North Eastern Province indicates a greater prevalence of the virus than was expected. WNV has not been previously reported in mosquitoes from these 2 villages, and study results imply regional variance in infection rates. Further studies may elucidate a difference between these 2 villages with regard to resident reservoirs (birds) or undiscovered amplifying hosts, especially if data are collected during outbreak conditions of flooding and mosquito proliferation. The spatial overlap of human population density with mosquito abundance (Figure 4) and the proximity of humans to infected mosquitoes (Figure 5), suggest that RVFV and WNV transmission during epizootic/epidemic periods could be high in both villages. Additional exposure-modifying factors, including the relative contribution of aerosol transmission of RVFV and the effects of housing construction, sleep and work habits, and the role of personal protective measures need to be further elucidated (*2*).

Our study has several limitations. Mosquito sampling during the outbreak was not stratified, and pooling of collected mosquitoes was not randomized (*39*). Mosquito sampling was conducted only at homesteads where specific animals, those known to be reservoirs of RVFV, were housed closely with humans. This sampling method may have underestimated the WNV MIR detected. This type of targeted sampling, however, can provide earlier detection of arboviruses and greater understanding of transmission and maintenance factors of these viruses (*39*). Although only 920 mosquitoes were tested for WNV and RVFV, a fraction of the total mosquito population collected, it has been shown that testing of mosquito pools versus testing of all samples can yield suitable results, thereby conserving time and resources (*39**,**40*). The choice of screening pools for arboviruses offers many benefits, especially during an outbreak. The potentially limiting factors of cost and time are avoided, while mosquito positivity is accurately identified (*33**,**39*).

In conclusion, we found high MIR for RVFV and WNV for many mosquitoes, some potentially efficient vectors, in our study region during the 2006–07 RVF outbreak in northeastern Kenya. MIRs did not differ between villages, although RVFV pool positivity and human seroprevalence (as measured in a previous homestead-based study during an interepidemic period) were higher in the rural village of Gumarey (*2*). Our data demonstrate the local abundance of mosquitoes infected with arboviruses in Kenya and highlights simultaneous arbovirus circulation. A greater understanding of how these arboviruses are maintained in nature will improve targeted prevention in regions where disease is endemic and curtail introduction to new areas. Our current inability to quickly detect arboviral infections in endemic communities has led to inaccurate risk assessments, underdiagnosis of clinical cases, and ineffective control measures. Better detection methods in vector, animal, and human populations and recognition of arboviral risk zones and circulation may alter current perceptions about these diseases. These methods could also lead to improved surveillance and better estimates of the true impact of arboviral disease on animal and human populations.
